# Case report on postmortem fentanyl measurement after overdose with more than 67 fentanyl patches

**DOI:** 10.1007/s11419-021-00598-3

**Published:** 2021-10-06

**Authors:** L. E. J. Peeters, I. T. Vleut, G. E. Tan, E. A. Croes, C. Bethlehem

**Affiliations:** 1 Department of Hospital Pharmacy, Erasmus MC, University Medical Center Rotterdam, Postbus 2040, 3000 CA Rotterdam, The Netherlands; 2 Department of Internal Medicine, Erasmus MC, University Medical Center Rotterdam, Rotterdam, The Netherlands; 3 Public Health Service (GGD) Region Utrecht, Utrecht, The Netherlands; 4 Trimbos Institute, Utrecht, The Netherlands

**Keywords:** Drug overdose, Toxicology, Humans, Fentanyl, Tandem mass spectrometry, Postmortem

## Abstract

**Purpose:**

Fentanyl is an analgesic that is frequently prescribed, which resulted in non-intentional as well as intentional misuse and deaths. Here, we present a postmortem case of a patient who clearly died of a fentanyl overdose due to an extensive number of fentanyl patches combined with oral intake of fentanyl and cocaine. We aimed to show how postmortem analysis can be used to interpret postmortem fentanyl concentrations in unique cases like the one we present.

**Case description:**

A 23-year-old male was found dead in his bedroom with 67 non-prescribed patches of fentanyl on his body. In the room, there also were fentanyl tablets of 100 µg and cocaine powder, which had possibly also been taken by the deceased. To confirm the cause of death, urine and subclavian blood were retrieved to perform a standard postmortem toxicology screening. The toxicological screening revealed the presence of several drugs, including cocaine, fentanyl, lidocaine and paracetamol. Further analysis of the quantitative postmortem values of fentanyl with ultra-performance liquid chromatography-tandem mass spectrometry revealed a fentanyl concentration of 57.9 µg/L. Considering several issues around postmortem drug analyses, this value seemed to be in line with concentrations found in previously reported postmortem cases.

**Conclusion:**

We were able to confirm the expected cause of death with an extensive toxicological screening in combination with the circumstantial evidence. We identified fentanyl as most important cause for the fatal outcome in this specific case and simultaneously contributed to the limited availability of knowledge on postmortem fentanyl concentrations.

## Introduction

The use and misuse of opioids has increased tremendously over the past decades, which caused opioids to be the primarily cause of death in drug overdoses in the United States [1]. One of the reasons that led to this, was the shift to an increased use of synthetic opioids in which fentanyl was identified as most important factor.

The increased popularity of fentanyl is mainly caused by the availability of specific formulations, such as fentanyl patches, which have several advantages. First of all, fentanyl is a synthetic opioid agonist with a high affinity for the µ-receptor, which makes it a strong analgesic. Second, fentanyl patches are a relatively patient-friendly formulation for patients with acute and chronic pain. Also, fentanyl has the ability to pass blood–brain barrier due to its lipophilic character. Besides, transdermal fentanyl is associated with fewer adverse gastrointestinal events than other opioid agonists, which are often given orally [2]. Fentanyl patches achieve a steady-state of fentanyl within 24 h after application and slowly release fentanyl over a period of 48–72 h.

However, these advantages also contribute to some major disadvantages. Especially, the addictive effects of the patches lead to considerable problems, as addiction and withdrawal problems lead to prolonged use, misuse and sometimes overdoses. Furthermore, patients as well as physicians are unaware of the risks of fentanyl, which could lead to unintentional deaths if the dose is not carefully titrated or if products with an unknown dose are used. Also, the amount of fentanyl that will lead to death remains undeterminable [3]. The establishment of lethal concentrations is complicated by drug tolerance after long term use on the one hand and by a large variability in plasma concentrations between patients on the other hand [4]. Although the awareness of these problems has increased in the last few years, this did not prevent further deaths due to fentanyl. Recent studies have even reported an increasing number of fentanyl intoxications and deaths by fentanyl overdoses, both intentional and unintentional [5–7].

Because of this increase in fentanyl intoxications, determination and interpretation of postmortem fentanyl concentrations is becoming more and more important. One of the first steps to determine the cause of death by opioids is a toxicological screening. This screening provides insight into which drugs are present at the time of death and gives an estimation of the amount of drugs that is present by measuring semi-quantitative concentrations. The screening cannot be used to determine exact quantitative drug values and in most postmortem cases these values are not required. However, in some cases it can be useful to quantify postmortem drug concentrations in order to further establish the lethal dose of fentanyl for instance. When measuring postmortem quantitative concentrations, careful interpretation of these values is necessary as postmortem fentanyl concentrations do not resemble ante mortem concentrations [8]. Also, there is a minimal availability of postmortem cases in which opioid use is known, which makes comparison between cases hard.

Here, we present a postmortem case of a patient who clearly died of a fentanyl overdose due to an extensive number of fentanyl patches combined with oral intake of fentanyl and cocaine. These specific features make this case unique and an addition to previously described fentanyl postmortem cases. We described how postmortem analysis was used in this specific case to interpret postmortem fentanyl concentrations and how this can complement the determination of the cause of death.

## Case description

A male person (age 23 years, length 184 cm and weight approximately 80 kg) was found dead in his own bed while lying on his back with an extensive number of fentanyl patches applied to his upper body. Circumstantial evidence including empty packages of various fentanyl patches and tablets found around the man, suggested a non-natural cause of death caused by an intentional fentanyl overdose. Because of this, a forensic doctor was notified to examine the body. The time of death was established around 6:00 a.m., approximately 12 h before the examination of the body took place, which was around 6:00 p.m. It was likely that the patches were applied before going to bed around midnight.

The total dose of fentanyl from the patches was calculated as 1310 µg/hour and included 39 patches of 12.5 µg/hour, 24 patches of 25 µg/hour, 3 patches of 50 µg/hour and one patch of 75 µg/hour. It was suspected that 30 tablets of 100 µg fentanyl and an unknown amount of cocaine were used simultaneously with the patches. This was derived from the presence of white powder near the patient as well as the presence of empty blisters (effentora^®^). These drugs were not prescribed to the man and most likely ordered via the internet. Both the use of cocaine and fentanyl were demonstrated in urine by means of a positive multi-drug dipstick. No signs of physical injuries were found on the body by the forensic doctor, suggesting a mixed-drug intoxication as cause of death. According to the family the man was physically healthy, but he did have a history of negative thoughts with suicidal manifestations for which he was previously admitted to acute psychiatric crisis care. Police investigation revealed that cetirizine and alprazolam were available to him, as they were found in prescribing records. This was in accordance with the alprazolam that was found at the man’s house as Ksalol^®^ 1 mg.

Urine and blood samples were collected according to the standard local procedures, to obtain more information about the role of substances in the cause of death. Urine was sampled by means of a puncture and subclavian blood was collected, since femoral blood could not be sampled. No autopsy was performed.

The urine and blood samples were sent to the Erasmus Medical Center to perform a toxicological screening, as part of a standard procedure of the inspection by the forensic doctor recorded in a local protocol. The standard toxicological screening in urine includes drugs of abuse (DOA) test screening (Abbott Diagnostics, Fremont, USA) for amphetamines, barbiturates, benzodiazepines, cocaine, cannabinoids, opiates and methadone, using the Abbott Architect C4000 analyzer (Abbott Diagnostics). The same analyzer was used to determine the ethanol concentration in the blood sample (plasma) using an immunoassay test (Abbott Diagnostics). For the toxicological screening in blood (plasma) samples, two applications of Waters were used [9, 10], using a UPLC Waters Acqity system (Waters Corporation, Milford, USA) coupled to a triple quadrupole mass spectrometer (Waters Xevo TQ-S micro 1), performing qualitative full scan MS and targeted multiple reaction monitoring (MRM)-based screening, with a library containing over 1200 compounds.

The drugs of abuse screening in the urine sample showed a positive result for the presence of cocaine. The toxicological screening in blood identified the presence of fentanyl and several other compounds including cocaine and metabolites, alprazolam, lidocaine and paracetamol of which the last three in sub-therapeutic concentrations. The concentration of benzoylecgonine was approximately 1 mg/L. Concentrations of cocaine and ecgonine methyl ester were not quantified but lower than 1 mg/L. The ethanol concentration measured by means of the immunoassay was low with a value of 0.22 g/L. In addition to these standard analyses, fentanyl concentrations in blood were quantified by means of a specific validated ultra-performance liquid chromatography–tandem mass spectrometry (UPLC-MS/MS) method (Waters Corporation) to measure fentanyl, sufentanil, cefazolin, doxapram and keto-doxapram [11]. With this method a postmortem fentanyl concentration of 57.9 µg/L was found, which was in line with earlier describe postmortem cases due to large amounts of fentanyl patches. In most of these cases the postmortem concentrations were > 50 µg/L as can be seen in Table 1. The toxicological screening in combination with the circumstantial evidence confirmed the expected cause of death due to a fentanyl overdose as most important factor.

**Table 1:** Comparison of postmortem concentrations fentanyl in several different post mortem cases after the use of fentanyl patches

Source	Sample collection	Type of overdose by means of fentanyl patches	Doses (mcg/h)	Time between dead and sampling	Postmortem concentration fentanyl (mcg/L)
Our case	Subclavian blood	Mixed-drug overdose + possible oral fentanyl intake	1310 + 3000 mcg oral fentanyl	± 12 h	57.9
Reiter et al.[8]	Femoral blood	Normal dose	150	± 24 h	21.9
Wiesbrock et al. [24]	Femoral blood	Overdose	1350	192 h	94.9
Andresen et al. [16]	Peripheral blood	Overdose	700	Unknown	65.7
			1000		127
Nara et al. [25]	Femoral blood	Mixed-drug overdose	350	± 24 h	51
Lung et al. [26]	Peripheral blood	Mixed-drug overdose	250	Unknown	53
			200		58
			75		50
Geile et al. [5]	Femoral blood	Mixed-drug overdose	150	Unknown	12.6
			300		29.3

## Discussion

This case describes a young man who died after an intentional overdose with fentanyl patches, which was confirmed by toxicological postmortem analyses. Also, the found fentanyl concentration seems to be in line with the number of fentanyl patches that were applied. The clear overdose with fentanyl patches in combination with the place of blood sampling and the possible use of several other substances, makes this case unique.

In postmortem cases a toxicological screening in blood and a DOA-test in urine are important tools to screen for drugs to elucidate the possible cause of death. These methods are used in addition to a multi-drug dipstick that is used at the crime scene and not only confirm results from the dipstick, but can also identify other substances that are not included in the dipstick. In our case the presence of fentanyl was detected at the crime scene by means of a positive multi drug dipstick in urine. These results were confirmed with the toxicology screening in blood.

On top of identifying substances, both the toxicological screening and DOA-test can provide semi-quantitative values of some substances. In most cases semi-quantitative values are sufficient and a more accurate quantitative measurement is not necessary while it will not provide any additional information to the cause of death of most postmortem cases. However, in cases with mixed drug overdose, like the one we described, quantitative analysis can be used to clarify and support the cause of death by one of the used substances. To explain the fentanyl concentration found in our case, several of these issues are mentioned hereafter.

After death the circulation in the body stops causing cell death, leakage of fluids and thereby postmortem drug redistribution [12, 13]. Furthermore, the composition of the body fluids change, among others, due to hemolysis [13]. These changes will extend over time, which makes the time after death an important factor to take into account during the interpretation of postmortem drug concentrations. To evaluate fatal drug concentrations more accurately, it is recommended to collect samples from various organs and tissues. Another reason that can result in the variability of postmortem concentrations, are the properties of the drug itself.

Finally, the location of sampling can be of influence on the height of the drug concentration. The femoral vein is preferred for sampling while this vein is less susceptible to postmortem changes [14]. In our case, subclavian blood was sampled, as femoral blood was not available. It is assumed that drug concentrations measured in subclavian blood are higher than concentrations in femoral blood. This was also seen by Molina et al*.* who found a 1.3 fold difference between the concentration of substances measured in subclavian blood compared to that in femoral blood [15]. Due to this difference and the fact that femoral blood is used most often in postmortem cases, it was difficult to compare our measured fentanyl concentration with previous cases.

In Table 1 we tried to compare postmortem fentanyl concentrations reported in several studies after the use of fentanyl patches taking into account the earlier mentioned issues. The measured fentanyl concentration (quantitative value) in our case was almost never seen after a normal dose of fentanyl when looking at previously reported measurements which can be seen in Table 1 [16, 17]. Although large interpatient variability was to be expected due to all the above mentioned issues [8], postmortem fentanyl concentrations higher than 50 µg/L are mostly seen after fatal fentanyl intoxications [17, 18]. This also seems to be independent of the administered dose of fentanyl patches [16], suggesting the amount of patches will only speed up the time that is needed to reach the lethal dose.

It should be noted that in our case fentanyl tablets were found in blisters. Therefore, it is possible that oral fentanyl was taken on top of the fentanyl patches. However, it is not expected that the found fentanyl concentration is largely influenced by the oral intake of fentanyl, because of two reasons. First, it is unlikely that the man dissolved 30 tablets in his mouth as recommended. Second, if all tablets were swallowed whole, not all of the fentanyl would have reached the blood stream, as normal absorption of one tablet after buccal use is around 50% [19].

A substance that could have contributed to the cause of death was cocaine. The presence of cocaine and metabolites were found in the toxicological screening. Cocaine is often used in combination with other drugs during suicide attempts, as it increases the confidence and energy levels due to neurotransmitter reuptake inhibition [20]. As mentioned before, the concentration benzoylecgonine was semi-quantified. This concentration was comparable with benzoylecgonine concentrations measured in impaired drivers (range < 0.01–2.0 mg/L [21]. When looking at the amount of cocaine and metabolites and the concentration of fentanyl, we found that the concentration of fentanyl is more associated with toxic values as compared to the amount of cocaine and benzoylecgonine. Therefore, we conclude that the amount of fentanyl was the most likely cause of death. However, as even small amounts of cocaine have the potential to be lethal, cocaine should be considered as a possible contributor to the cause of death.

Also, a small amount of 0.22 g/L ethanol was measured using an immunoassay method. There are several possible explanations for the presence of ethanol. First, the deceased may have ingested alcohol before he died. Second, the results from the immunoassay may have been influenced by increased lactic acid or lactate dehydrogenase (LDH) in the postmortem sample, causing the assay to show false positive ethanol concentrations [22, 23]. Finally, other gasses could have been produced postmortem, causing cross reactivity with the assay, which may have resulted in increased or false positive values of ethanol. Because of these issues the immunoassay results are normally confirmed in a quantitative analysis of volatiles by means of headspace gas chromatography with flame ionization detection (HS-GC-FID). However, in this case, the ethanol concentrations were relatively low and no intoxication was expected. Therefore, quantification of ethanol was considered less important and was not performed.

When looking at mixed-drug toxicity, slightly higher fentanyl concentrations were found in previously published cases [17]. However, it remains unknown how cocaine and ethanol influenced the fentanyl concentration in this specific case.

## Conclusion

We described a unique case in which extensive toxicological screening was used to confirm the cause of death after the use of fentanyl patches. Interpretation of postmortem fentanyl concentrations is difficult due to the large inter-individual variation in plasma concentrations and often due to a lack of crucial information, such as the location of sampling and the time between death and sampling. In this case, crucial information was available, from which we conclude that the measured fentanyl concentration of 57.9 µg/L tallies with the fatal outcome.
